# High proportion of recurrent germline mutations in the *BRCA1 *gene in breast and ovarian cancer patients from the Prague area

**DOI:** 10.1186/bcr1282

**Published:** 2005-07-19

**Authors:** Petr Pohlreich, Michal Zikan, Jana Stribrna, Zdenek Kleibl, Marketa Janatova, Jaroslav Kotlas, Jana Zidovska, Jan Novotny, Lubos Petruzelka, Csilla Szabo, Bohuslav Matous

**Affiliations:** 1 Department of Biochemistry and Experimental Oncology, First Faculty of Medicine, Charles University, Prague, Czech Republic; 2 Institute of Biology and Medical Genetics, First Faculty of Medicine, Charles University, Prague, Czech Republic; 3 Department of Oncology, First Faculty of Medicine, Charles University, Prague, Czech Republic; 4 Unit of Genetic Epidemiology, International Agency for Research on Cancer, Lyon, France

## Abstract

**Background:**

Germline mutations in the *BRCA1 *and *BRCA2 *genes have been shown to account for the majority of hereditary breast and ovarian cancers. The purpose of our study was to estimate the incidence and spectrum of pathogenic mutations in *BRCA1/2 *genes in high-risk Czech families.

**Methods:**

A total of 96 Czech families with recurrent breast and/or ovarian cancer and 55 patients considered to be at high-risk but with no reported family history of cancer were screened for mutations in the *BRCA1/2 *genes. The entire coding sequence of each gene was analyzed using a combination of the protein truncation test and direct DNA sequencing.

**Results:**

A total of 35 mutations in the *BRCA1*/*2 *genes were identified in high-risk families (36.5%). Pathogenic mutations were found in 23.3% of breast cancer families and in 59.4% of families with the occurrence of both breast and ovarian cancer. In addition, four mutations were detected in 31 (12.9%) women with early onset breast cancer. One mutation was detected in seven (14.3%) patients affected with both a primary breast and ovarian cancer and another in three (33.3%) patients with a bilateral breast cancer. A total of 3 mutations in *BRCA1 *were identified among 14 (21.4%) women with a medullary breast carcinoma. Of 151 analyzed individuals, 35 (23.2%) carried a *BRCA1 *mutation and 9 (6.0%) a *BRCA2 *mutation. One novel truncating mutation was found in *BRCA1 *(c.1747A>T) and two in *BRCA2 *(c.3939delC and c.5763dupT). The 35 identified *BRCA1 *mutations comprised 13 different alterations. Three recurrent mutations accounted for 71.4% of unrelated individuals with detected gene alterations. The *BRCA1 *c.5266dupC (5382insC) was detected in 51.4% of mutation positive women. The mutations c.3700_3704del5 and c.181T>G (300T>G) contributed to 11.4% and 8.6% of pathogenic mutations, respectively. A total of eight different mutations were identified in *BRCA2*. The novel c.5763dupT mutation, which appeared in two unrelated families, was the only recurrent alteration of the *BRCA2 *gene identified in this study.

**Conclusion:**

Mutational analysis of *BRCA1/2 *genes in 151 high-risk patients characterized the spectrum of gene alterations and demonstrated the dominant role of the *BRCA1 *c.5266dupC allele in hereditary breast and ovarian cancer.

## Introduction

Breast cancer (BC) is the most common malignancy affecting western women. About 5% to 10% of all BC cases are due to inheritance of a susceptibility allele, consistent with transmission in an autosomal dominant fashion, and a substantial proportion of these are due to germline mutations of the two major highly penetrant cancer susceptibility genes, *BRCA1 *(OMIM, 113705; GenBank, U14680.1) [1,2] and *BRCA2 *(OMIM, 600185; GenBank, U43746.1) [3-5]. Hereditary BC is characterized by an early age of onset, high incidence of bilateral disease and frequent association with ovarian cancer (OC). An increased incidence of other malignancies, such as colorectal, prostate and pancreatic cancer is also observed among *BRCA1/2 *mutation carriers [6-8]. The proportion of described mutations in *BRCA1 *relative to *BRCA2 *varies between populations. With the exception of a strong *BRCA2 *founder effect in Iceland [9], however, *BRCA1 *mutations are generally more frequently reported. In the majority (>80%) of families with BC and OC, the diseases are linked to the *BRCA1 *gene. Conversely, in the majority (>75%) of families with male and female BC, the disease is linked to *BRCA2*. Among families with female BC only, proportions of diseases due to mutations in *BRCA1*, *BRCA2 *and other genes are similar [10].

A large number of distinct mutations, polymorphisms and genetic variants of uncertain significance in the *BRCA1 *and *BRCA2 *genes is described in the Breast Cancer Information Core Database (BIC Database) [11]. The majority of mutations known to be disease causing result in a truncated protein due to frameshift, nonsense or splice site alterations. The spectrum of mutations varies between populations, with some showing a high frequency of unique mutations, for example in Italy [12,13], whereas a small number of founder mutations is more common in other ethnic groups. Notably, a single founder mutation in *BRCA2 *(c.771_775del5; commonly referred to as 999del5) accounts for the majority of hereditary cancer cases in Iceland [9], and three ancestral mutations (c.68_69delAG and c.5266dupC in *BRCA1 *and c.5946delT in *BRCA2*; 185delAG, 5382insC and 6174delT, respectively) were identified in the vast majority of families with a history of BC and OC in Ashkenazi Jews [14]. Population specific mutations have also been described in the Netherlands [15], Sweden [16], France [17], Spain [18] and other countries [19]. Two *BRCA1 *founder mutations, c.5266dupC and c.181T>G (300T>G), occur most frequently in countries of Central and Eastern Europe [20-25], including the Czech Republic [26].

The aim of this study was to estimate the incidence, spectrum and possible clustering of disease phenotypes associated with *BRCA1 *and *BRCA2 *mutations in the Prague area and Central Bohemia. The analysis was performed in families with a history of BC/OC and in high-risk patients not selected on the basis of their family history of cancer.

## Materials and methods

### Patients and families

Women with BC or OC considered to be at high risk of carrying a *BRCA1 *or *BRCA2 *mutation were selected for genetic testing between 1998 and 2003 at the Department of Oncology and at the Department of Gynecology and Obstetrics of the First Faculty of Medicine Charles University in Prague. The testing was performed immediately after confirming the pathologic diagnosis. All patients had Czech ancestries and were living in the Prague area and Central Bohemia. Patients were selected from cancer families that met the following criteria in first- or second-degree relatives: two cases of either BC diagnosed before the age of 50 or OC diagnosed at any age; and three or more cases of breast or ovarian cancer diagnosed at any age. A total of 60 families had a history positive for BC only (HBC families), 4 families had OC only (HOC families), and 32 families had both breast and ovarian cancer (HBOC families). Genetic material for analysis was obtained from the youngest affected individual from each family. Genetic testing was further offered to patients diagnosed with BC before the age of 36 (31 women) or with bilateral BC before the age of 51 (3 women) and patients with both primary breast and ovarian cancer (7 women) or medullary breast carcinoma diagnosed at any age (14 women), regardless of absence of reported family history of cancer (non-familial patients). All women in the study gave their informed consent prior to genetic testing. The protocol of investigation was approved by the Ethical Committee at the First Faculty of Medicine.

### DNA and RNA isolation

Genomic DNA was isolated from EDTA blood samples using the Wizard genomic DNA purification kit (Promega, Madison, USA), according to the manufacturer's instructions. Total RNA was obtained from peripheral blood lymphocytes and reverse transcribed into cDNA as described [27].

### Screening for *BRCA1 *and *BRCA2 *mutations

Most disease-associated mutations lead to premature termination of protein translation. Mutations are classified as deleterious if they truncate either the BRCA1 protein at least 10 amino acids from the C-terminus or the BRCA2 protein at least 110 amino acids from the C-terminus [28]. The nomenclature of mutations used is according to den Dunnen and Paalman [29], with nucleotides numbered from the A of the ATG translation initiation codon of GenBank reference sequences U14680.1 for *BRCA1 *cDNA and U43746.1 for *BRCA2 *cDNA. Original designations for *BRCA1/2 *mutations commonly referred to in the literature are included for ease of cross-referencing.

Mutational analysis was carried out by the protein truncation test (PTT) and direct DNA sequencing. The entire coding region of *BRCA1 *and *BRCA2 *was divided into overlapping fragments with sizes of 880 to 1569 bp and amplified by PCR. The PTT assay was used for pre-screening of amplified fragments; the final analysis of identified gene alterations was done by sequencing appropriate PCR fragments. Large exons (exon 11 of *BRCA1 *and exons 10 and 11 of BRCA2) were amplified on genomic DNA (exon 11 of *BRCA1 *and *BRCA2 *in three and four fragments, respectively; exon 10 of *BRCA2 *as a single fragment), whereas the remaining coding exons were amplified by two-step PCR (nested PCR) from cDNA (exons 2 to 10 and 12 to 24 of *BRCA1 *and exons 2 to 9 of *BRCA2 *as single fragments; exons 12 to 27 of *BRCA2 *in two fragments). Primer sequences used for amplification of *BRCA1 *and *BRCA2 *fragments were as described [16,30,31].

Amplifications were performed in 12.5 μl reaction mixtures containing PCR buffer (10 mM Tris-HCl, pH 8.3, 50 mM KCl, 1.5 mM MgCl_2_), 0.2 mM dNTPs, 0.4 μM of each primer, 0.5U of LA Taq DNA polymerase (Takara Shuzo Co., Shiga, Japan) and 30–50 ng of genomic DNA. Following initial denaturation (at 93°C for 2 minutes), 32 cycles (at 93°C for 1 minute, at 58°C for 1 minute, and at 72°C for 4minutes) and final extension (at 72°C for 5minutes) were performed. In the nested PCR procedure, a 2 μl aliquot of the reverse transcription reaction was used in the first round of amplification with external primers. A 1 μl aliquot of the first PCR reaction was removed and used as a template with internal primers in the second round of amplification. Reaction conditions and cycling parameters were as described above.

PTT-analysis was carried out by incubating PCR fragments (0.5 μl) in the TnT/T7 coupled transcription/translation system (Promega, Madison, USA) containing 0.5 to 1.0 μCi of L-[^35^S]methionine (Amersham Biosciences, Buckinghamshire, UK) for 90 minutes at 30°C in a total volume of 3 μl. Labeled protein products were analyzed on 12% SDS/polyacrylamide minigels (Bio-Rad Laboratories, Hercules, USA). The gels were fixed and prepared for fluorography by washing in Amplify (Amersham Biosciences, Buckinghamshire, UK). Dried gels were exposed for 24 to 48 h to X-ray film at -80°C.

### DNA sequencing

PCR products that gave rise to truncated proteins by PTT analysis were gel purified and directly sequenced in forward and reverse directions using the BigDye 3.1 terminator cycle sequencing kit in a model 310 automated DNA sequencer (Applied Biosystems, Foster City, USA). Mutations detected by RNA-based analysis were confirmed by DNA sequencing. Each identified sequence alteration was confirmed by the analysis of a second blood sample.

Frequently occurring mutations at the beginning and at the end of *BRCA1 *(c.68_69delAG, c.181T>G and c.5266dupC) were identified by sequencing of RT-PCR fragments corresponding to exons 2 to 8 and 18 to 24. Sequence analyses of amplified genomic fragments containing the end of exon 11 in *BRCA1 *and exons 17 and 20 in *BRCA2 *were done as direct tests for other recurrent variants (c.4034delA in *BRCA1 *and c.7910_7914del5 and c.8537_8538delAG in *BRCA2*) known to occur in the Czech Republic and in populations of Central and Eastern Europe. In addition, in families with negative results from mutation analysis and with a strong history of cancer (families with three or more BC and/or OC cases), short exons of *BRCA1 *and *BRCA2 *were further screened for mutations by direct sequencing of RT-PCR fragments or by radioactive heteroduplex analysis following the protocol described by Gayther *et al*. [32] and Serova *et al*. [33].

### Statistical analysis

Differences in mutation frequencies among groups of analyzed families were statistically evaluated by the Chi-square test.

## Results

Mutation analysis was performed in 96 women from BC/OC families and in 55 non-familial patients. Analysis revealed 44 pathogenic cancer predisposing mutations, 6 of which have been previously reported elsewhere [27]. Within 151 analyzed individuals, 35 (23.2%) carried a *BRCA1 *mutation and 9 (6.0%) a *BRCA2 *mutation.

The *BRCA1 *mutations comprised 13 distinct alterations distributed widely across the coding sequence of the gene (Table 1). Twelve gene alterations caused a premature protein termination: eight were frame-shift alterations, with the majority of small deletions and insertions occurring in stretches of mononucleotide or dinucleotide repeats, and four were nonsense mutations. The c.181T>G mutation leading to a substitution of conserved cysteine 61 with glycine (p.Cys61Gly) in the RING finger domain of the BRCA1 protein was the only missense mutation identified in the gene.

Of the 10 *BRCA1 *mutations observed only once in our series, the c.1747A>T nonsense mutation is a novel gene alteration (not reported to the BIC by June 2004) found in family 397 with two cases of OC diagnosed at the ages of 39 and 43. Four additional mutations (c.1016delA, c.3331C>T, c.1127delA and c.2263G>T) belong to rare gene alterations (with one to four entries in the BIC database), whereas the others (c.68_69delAG, c.1687C>T, c.2411_2412delAG, c.3756_3759delGTCT and c.4165_4166delAG) occur frequently in various European regions, including Central Europe.

Three recurrent mutations were found in 25 (71.4%) of the 35 women with detected alterations in *BRCA1*. The mutation c.5266dupC (5382insC) was a highly prominent mutation detected in 18 patients, which accounted for 51.4% of all identified alterations in *BRCA1*. The mutation c.3700_3704del5 found in four families was the second most commonly identified alteration, which contributed to 11.4% of mutations detected in *BRCA1*. The mutation c.181T>G (300T>G; p.C61G) identified in three families comprised 8.6% of detected mutations.

The *BRCA2 *mutations included eight different gene abnormalities (Table 2). All alterations were localized to exon 11 and led to a truncated protein product: five were frameshift alterations and three were nonsense mutations. The mutation c.3939delC is a novel frameshift mutation, which results in a termination of translation at codon 1313. This mutation was detected in family 348 with two cases of OC diagnosed at the ages of 46 and 58. Both the c.3076A>T nonsense mutation (occurring in conjunction with the c.3075G>T missense mutation) and the frameshift mutation c.5238dupT belong to rare, infrequently reported gene alterations. Other identified mutations (c.2808_2811delACAA, c.3975_3978dupTGCT, c.5645C>A and c.5682C>G) occur frequently throughout Western Europe.

Recurrent mutations represented over two-thirds of all the *BRCA1 *mutations identified in this series. By contrast, the alterations in *BRCA2 *were mostly unique. The c.5763dupT mutation, which causes a premature termination of translation at codon 1923, appeared in two families and was the only recurrent alteration of the *BRCA2 *gene identified in this study. The mutation was first detected in family 67 with breast and ovarian cancer diagnosed before the age of 50. In the second unrelated family (F-327), only one affected woman who developed BC at the age of 32 was found. This gene alteration has since been reported once in Western Europe, as indicated in the BIC database.

Table 3 shows the prevalence of mutations in BC/OC families and in non-familial risk patients who had no reported family history of cancer. Pathogenic mutations were revealed in 35 (36.5%) of the 96 analyzed families. The incidence of mutations differed significantly between HBC (14/60; 23.3%) and HBOC (19/32; 59.4%) families (p = 0.0006). Two mutations were found in four HOC families.

Non-familial patients included a group of 31 women diagnosed with BC between ages 22 years and 35 years without history of BC or OC in their family. Pathogenic germline mutations in predisposing genes were detected in four (12.9%) women. Three mutations were identified in *BRCA1 *(c.68_69delAG, c.181T>G and c.5266dupC) and one in *BRCA2 *(c.5763dupT). Screening of seven patients with both breast and ovarian cancer and no family history identified one mutation (14.3%) in the *BRCA1 *gene (c.5266dupC). The mutation c.5266dupC was also detected in a group of three patients (33.3%) with bilateral BC.

A high incidence of medullary carcinoma has been reported among women with *BRCA1*-associated BC [34]. We performed the analysis of *BRCA1 *and *BRCA2 *genes in 14 women with this histological tumor subtype and found three truncating mutations (21.4%) in exon 11 of the *BRCA1 *gene. The c.3331C>T nonsense mutation was detected once, whereas the c.3700_3704del5 frameshift mutation was identified in two cases. No alteration in the *BRCA2 *gene was found among these analyzed patients.

## Discussion

In our study, 35 mutations in the *BRCA1 *and *BRCA2 *genes were detected in 96 BC/OC families. In addition, we found four pathogenic mutations in patients with early onset BC, one mutation in a case of a bilateral BC, one mutation in a woman with both BC and OC, and three mutations in women with a medullary breast carcinoma. The majority of mutations identified in our study lead to protein truncations. Although short coding exons of both *BRCA1 *and *BRCA2 *were analyzed by either direct sequencing or heteroduplex analysis, only the *BRCA1 *c.181T>G (c.300T>G; p.C61G) missense mutation and *BRCA2 *c.3075G>T (p.K1025N) missense alteration (present in conjunction with the K1026X nonsense mutation) of unknown clinical significance were observed. One novel mutation was found in *BRCA1 *(c.1747A>T); two novel mutations were identified in *BRCA2 *(c.3939delC and c.5763dupT).

The prevalence of inherited *BRCA1/2 *mutations observed in different studies varies according to the ethnic origin of analyzed individuals, criteria of selection for genetic testing and techniques used for mutational screening. Although the techniques we applied in our study compromised our ability to detect missense mutations in regions of the *BRCA1/2 *genes screened only by PTT, at present the majority of reported missense alterations are difficult to interpret with respect to potential clinical significance, as the effect of the amino acid substitution on protein function is not yet well understood. Conversely, most protein truncating *BRCA1/2 *mutations are presumed to be pathogenic, thereby permitting women harboring such mutations to be provided appropriate clinical counseling and management. Our study may not have detected large genomic rearrangements encompassing regions outside the primer sets used for RT-PCR amplification. On the other hand, in the absence of rapid degradation of aberrant transcripts by nonsense-mediated mRNA decay [35], any rearrangements encompassing the exons within the regions amplified from cDNA would have been observed as aberrantly sized PCR products, as has also been found in other studies [36]. Further, the prevalence of genomic rearrangements varies between populations. To our knowledge, large deletions and rearrangements in *BRCA1/2 *genes have not been reported in countries of Central and Eastern Europe. In our series, analysis of truncated RT-PCR products and sequencing of corresponding genomic fragments identified one case with the rearrangement that involved exons 21 and 22 of the *BRCA1 *gene (Zikan, unpublished results). Despite the limitations of our approach, the prevalence of mutations in our group of high-risk families was comparable to that observed in Central Europe [21,23,26]. Further, the distribution of germline *BRCA1/2 *mutations in our high-risk families is consistent with a higher prevalence in the context of OC [10]; mutations were detected significantly more frequently in HBOC and HOC families than in HBC families (58.3% of 36 versus 23.3% of 60; p = 0.0006).

Interestingly, both *BRCA1 *and *BRCA2 *mutationswere more prevalent in families with OC (Table 3). Of the 27 *BRCA1 *mutations detected in high-risk families, 11 were present in HBC families (18.3%), whereas 16 were identified in HBOC and HOC families (44.4%). Of the eight mutations detected in *BRCA2*, three were present in HBC families (5%), relative to five in HBOC and HOC families (13.9%). This observation is in contrast to observations in larger series of examined families, where the risk of OC was significantly greater among women with *BRCA1 *mutations compared to women with *BRCA2 *mutations [10,37]. The higher prevalence of *BRCA2 *mutations among families with OC in our study may be due to the preponderance of mutations identified in the ovarian cancer cluster region, and lend support to the increased risk of OC suggested to be conferred by mutations within this region relative to other *BRCA2 *mutations [38,39].

Gayther *et al*. [40] have reported that mutations in the 3' third of the *BRCA1 *gene are associated with a lower proportion of ovarian cancer. The border for this phenotype correlation was located at exon 13, between codons 1435 and 1443. Further studies provided proof for this genotype-phenotype correlation [16,41], although other authors failed to replicate this observation for *BRCA1 *mutations [17,20]. Despite the higher occurrence of ovarian cancer in high-risk families with mutations located in exons 2 to 12, the relative frequency (12/37 cancer cases; 32.4%) did not differ significantly in our study from the frequency of ovarian cancer (11/51 cancer cases; 21.6%) in patients with mutations in exons 14 to 24 (p = 0.25).

The surprising finding of our investigation was a high predominance of recurrent mutations in the *BRCA1 *gene, which contributed to a substantial proportion of hereditary BC and OC cases. The three repeatedly occurring mutations in *BRCA1 *were detected in more than 56% of women with identified alterations in *BRCA1/2 *genes and in more than 71% of women with alterations in *BRCA1*. The most frequent mutation was c.5266dupC (5382insC) found in 15 (15.6%) of 96 analyzed BC/OC families and in 18 (40.9%) of 44 *BRCA1/2 *mutation positive patients. The occurrence of this mutation is comparable to that found in Polish [22,23,25,42] and Russian [20] populations, but significantly higher than that described in Germany [43] and Austria [21]. The c.5266dupC mutation is the most prevalent *BRCA1 *alteration in Europe and a geographic distribution of this mutation is consistent with its Baltic origin [19]. The c.5266dupC allele also occurs at a high frequency (0.11%) in the Ashkenazi Jewish population [14], although the 18 Czech patients carrying this gene defect did not report an Ashkenazi Jewish heritage. The other two recurrent mutations detected in our group of patients, c.3700_3704del5 and c.181T>G (300T>G), also belong to gene alterations that have been repeatedly detected in the Central European population [23-26].

The *BRCA1 *c.5266dupC and c.181T>G mutations are prevalent in Poland and in the Czech Republic, although spectra of mutations display significant differences in these countries [26,42]. The mutation c.4034delA (4153delA), contributing to 9.8% of mutations identified in *BRCA1 *in Poland [42], did not occur either in our or in Moravian families [26]. We identified 10 unique mutations in *BRCA1*, which suggests that the spectrum of alterations in this gene is more heterogeneous than that reported in Poland [42].

In contrast to the *BRCA1 *gene, there were few recurrent mutations within *BRCA2*. With the exception of the c.5763dupT mutation detected in two unrelated individuals, each alteration identified in *BRCA2 *was found in only one family.

In a set of pathogenic mutations in *BRCA1 *identified in Brno (Moravia) [26], the prevalence of the three most common mutations (c.5266dupC, c.3700_3704del5 and c.181T>G) was 37.3% (22/59), 13.6% (8/59) and 10.2% (6/59), respectively, whereas in our group of patients, the prevalence of these alterations was 51.4% (18/35), 11.4% (4/35) and 8.6% (3/35). Small variations in the mutation spectra observed in both studies may be caused by limited sample size and may also reflect differences in the groups of patients selected for genetic testing.

The occurrence of *BRCA2 *mutations was higher in Moravia (33%; 29/88 of mutation positive patients) than in our study (20.5%; 9/44 of mutation positive patients) and the spectrum of genetic alterations was completely different [26]. The c.7910_7914del5 (8138_8142del5) and c.8537_8538delAG (8765_8766deAG) alterations found in Brno in 7 (24.1%) of the 29 families with detected mutations in *BRCA2*, were not present in our group of patients. On the contrary, the mutation c.5763dupT, the only recurrent alteration of the *BRCA2 *gene identified in our study, was not found in Moravia. A high frequency of unique *BRCA2 *mutations may be characteristic of the examined Prague area and Central Bohemia, although the examination of a larger group of families is required to obtain valid results.

The incidence of *BRCA1/2 *mutations in a group of Czech women with early onset non-familial BC was 12.9% (4/31; Table 3), whichsuggests that the age at diagnosis in patients with a negative family history is an important indicator for the presence of a pathogenic mutation and lends support to the screening of *BRCA1/2 *genes in patients with early onset disease. In contrast to our findings, only 2% of non-familial patients had pathogenic germline *BRCA1 *and *BRCA2 *mutations in a group of patients in Great Britain who were diagnosed with BC at the age of 30 years or younger [44]. A similarly low frequency of mutations was found in non-familial patients in Spain and Iran [45,46]. In our study, all patients carried the most common, easily detectable mutations of *BRCA1 *or *BRCA2 *that prevail in this region and can be associated with early onset cancer. A larger set of patients with early onset BC is currently under investigation to determine the incidence of mutations in this risk group.

Medullary carcinoma of the breast is not common (2% to 3%) in patients with *BRCA2 *mutations and those with no known germline gene alteration [47]. In *BRCA1*-related BC, the incidence of typical medullary carcinoma was 19% (6/32) in a French study [34], 8% (4/49) in a Dutch study [48], but 0% in a Swedish series of 40 *BRCA1*-associated tumors [49]. In our study, one medullary carcinoma was identified in a group of 22 patients (4.5%) with *BRCA1*-related BC (F-252), whereas no tumor of this histological type was found in seven women with *BRCA2*-associated tumors. We did, however, find three germline *BRCA1 *mutations in a set of 14 patients (21.4%) with medullary breast carcinoma selected for examination regardless of the family history. These women did not belong to high-risk families and did not fulfill common criteria for genetic testing. One patient (Table 1; F-80) with a medullary carcinoma at age 50 had a sister affected with BC at age 54 and the other two (Table 1; F-305 and F-390) with medullary carcinomas at ages 38 and 42, respectively, were without a family history of breast or ovarian cancer. Our results are in agreement with studies of Eisinger *et al*. [34] who tested 18 cases of medullary carcinoma for mutations in *BRCA1 *gene and found two (11%) harboring *BRCA1 *mutations. Interestingly, these cases did not belong to high-risk families either. Indication of cases with medullary carcinoma for *BRCA1 *testing, regardless of the family history, may be helpful in mutation screening. Examination of a larger group of patients with this histological type of carcinoma is required to determine the importance of this morphological parameter more exactly.

## Conclusion

Mutational analysis of *BRCA1/2 *genes in 151 high-risk patients characterized the spectrum of gene alterations and demonstrated the dominant role of the *BRCA1 *c.5266dupC (5382insC) allele in hereditary breast and ovarian cancer. The pre-screening of high-risk patients for the presence of this allele, which accounted for more than 40% of all identified gene alterations, and pre-screening for the presence of other pathogenic, repeatedly occurring alleles (c.3700_3704del5 and c.181T>G), which contributed to about 16% of gene defects, could enable rapid detection of a high percentage of patients with hereditary cancer predisposition and, thus, reduce the cost of mutation analysis in the Czech population.

## Abbreviations

BC = breast cancer; BIC = Breast Cancer Information Core; HBC = hereditary breast cancer; HBOC = hereditary breast and ovarian cancer; HOC = hereditary ovarian cancer; OC = ovarian cancer; PCR = polymerase chain reaction; PTT = protein truncation test.

## Competing interests

The authors declare that they have no competing interests.

## Authors' contributions

PP and MZ contributed equally to this work.

## Figures and Tables

**Table 1:** Pathogenic germline *BRCA1 *mutations in breast and ovarian cancer patients from the Prague area

Family no.	Mutation description				Method of detection	No. of cancers in a family and age at onset		
	Exon	Traditional nomenclature	Approved nomenclature	Predicted effect		Breast cancer (bilateral)	Mean age at diagnosis	Other cancers (age at onset)

F-24	2	c.187_188delAG	c.68_69delAG	p.Glu23fsX39	Sequencing	1	35	Colon (54)
F-111	5	c.300T>G	c.181T>G	p.Cys61Gly	Sequencing	3	42	Stomach (51)
F-126	5	c.300T>G	c.181T>G	p.Cys61Gly	Sequencing	2	45	-
F-252	5	c.300T>G	c.181T>G	p.Cys61Gly	Sequencing	1	29	-
F-43	11	c.1135delA	c.1016delA	p.Lys339fsX340	PTT	2	41	Colon (50), lung (64)
F-361	11	c.1246delA	c.1127delA	p.Asn376fsX393	PTT	1	37	Ovarian (52, 54, 55)
F-21	11	c.1806C>T	c.1687C>T	p.Gln563X	PTT	1 (1)	46	Ovarian (43), melanoma (53)
F-397	11	c.1866A>T^a^	c.1747A>T^a^	p.Lys583X^a^	PTT	-	-	Ovarian (39, 43)
F-249	11	c.2382G>T	c.2263G>T	p.Glu755X	PTT	4	53	Ovarian (41, 54)
F-61	11	c.2530_2531delAG	c.2411_2412delAG	p.Gln804fsX808	PTT	3	49	-
F-80	11	c.3450C>T	c.3331C>T	p.Gln1111X	PTT	2	52	-
F-305	11	c.3819_3823del5	c.3700_3704del5	p.Val1234fsX1241	PTT	1	38	Leukemia (67), lung (65)
F-337	11	c.3819_3823del5	c.3700_3704del5	p.Val1234fsX1241	PTT	3	44	-
F-347	11	c.3819_3823del5	c.3700_3704del5	p.Val1234fsX1241	PTT	2	42	-
F-390	11	c.3819_3823del5	c.3700_3704del5	p.Val1234fsX1241	PTT	1	42	Lung (56), kidney (65)
F-164	11	c.3875_3878delGTCT	c.3756_3759delGTCT	p.Leu1252fsX1262	PTT	2 (1)	42	Ovarian (40, 43), stomach (?)
F-245	12	c.4284_4285delAG	c.4165_4166delAG	p.Ser1389fsX	HDA	2	38	Ovarian (44, 50), kidney (75)
F-15	20	c.5385dupC	c.5266dupC	p.Gln1756fsX1829	Sequencing	1	32	-
F-75	20	c.5385dupC	c.5266dupC	p.Gln1756fsX1829	Sequencing	1	44	Ovarian (?, ?)
F-152	20	c.5385dupC	c.5266dupC	p.Gln1756fsX1829	Sequencing	2	58	Ovarian (72)
F-185	20	c.5385dupC	c.5266dupC	p.Gln1756fsX1829	Sequencing	3	51	Colon (?)
F-187	20	c.5385dupC	c.5266dupC	p.Gln1756fsX1829	Sequencing	3	48	Ovarian (56)
F-194	20	c.5385dupC	c.5266dupC	p.Gln1756fsX1829	Sequencing	4	43	Ovarian (52)
F-201	20	c.5385dupC	c.5266dupC	p.Gln1756fsX1829	Sequencing	2	41	-
F-239	20	c.5385dupC	c.5266dupC	p.Gln1756fsX1829	Sequencing	3	46	Ovarian (41), stomach (?)
F-243	20	c.5385dupC	c.5266dupC	p.Gln1756fsX1829	Sequencing	3 (1)	42	-
F-261	20	c.5385dupC	c.5266dupC	p.Gln1756fsX1829	Sequencing	2	29	-
F-265	20	c.5385dupC	c.5266dupC	p.Gln1756fsX1829	Sequencing	3 (1)	52	Ovarian (?)
F-273	20	c.5385dupC	c.5266dupC	p.Gln1756fsX1829	Sequencing	3	36	Ovarian (42), colon (51, 56)
F-331	20	c.5385dupC	c.5266dupC	p.Gln1756fsX1829	Sequencing	1 (1)	31	Uterus (60), colon (64)
F-342	20	c.5385dupC	c.5266dupC	p.Gln1756fsX1829	Sequencing	2	50	Ovarian (41), colon (?)
F-368	20	c.5385dupC	c.5266dupC	p.Gln1756fsX1829	Sequencing	4 (2)	37	Ovarian (40), kidney 78)
F-370	20	c.5385dupC	c.5266dupC	p.Gln1756fsX1829	Sequencing	2	49	Ovarian (55)
F-385	20	c.5385dupC	c.5266dupC	p.Gln1756fsX1829	Sequencing	1	52	Ovarian (61), colon (83), melanoma (79)
F-387	20	c.5385dupC	c.5266dupC	p.Gln1756fsX1829	Sequencing	3 (1)	36	-

Position in cDNA is according to GenBank accession number U14680. ^a^Novel mutations. HAD, heteroduplex analysis; PTT, protein truncation test.

**Table 2:** Pathogenic germline *BRCA2 *mutations in breast and ovarian cancer patients from the Prague area

Family no.	Mutation description				Method of detection	No. of cancers in a family and age at onset		
	Exon	Traditional nomenclature	Approved nomenclature	Predicted effect		Breast cancer (bilateral)	Mean age at diagnosis	Other cancers (age at onset)

F-263	11	c.3036_3039delACAA	c.2808_2811delACAA	p.Lys936fsX958	PTT	3 (1)	41	-
F-279	11	c.3303G>T; 3304A>T	c.3075G>T; 3076A>T	p.Lys1025Asn; p.Lys1026X	PTT	1	50	Ovarian (47, 50)
F-348	11	c.4167delC^a^	c.3939delC^a^	p.Tyr1313fsX	PTT	-	-	Ovarian (46, 58), lung (?)
F-237	11	c.4203_4206dupTGCT	c.3975_3978dupTGCT	p.Ala1327fsX1331	PTT	2	38	Stomach (?)
F-298	11	c.5466dupT	c.5238dupT	pAsn1747fsX	PTT	1	41	Ovarian (59)
F-304	11	c.5873C>A	c.5645C>A	p.Ser1882X	PTT	3	61	Ovarian (49), colon (67)
F-49	11	c.5910C>G	c.5682C>G	p.Tyr1894X	PTT	2 (1)	45	Lung (?)
F-67	11	c. 5991dupT^a^	c.5763dupT^a^	p.Ala1922fsX1923^a^	PTT	1	48	Ovarian (49), esophagus (?)
F-327	11	c. 5991dupT^a^	c.5763dupT^a^	p.Ala1922fsX1923^a^	PTT	1	32	-

Position in cDNA is according to GenBank accession number U43746. ^a^Novel mutations. PTT = protein truncation test.

**Table 3:** Frequencies of *BRCA1 *and *BRCA2 *mutations in relation to the classification of patients and families

Classification	Number of patients/families	No. of mutations in *BRCA1 *(%)	No. of mutations in *BRCA2 *(%)	No. of *BRCA1 *+ *BRCA2 *mutations (%)
Cases without a family history of breast and ovarian cancer				
Breast cancer before 36	31	3 (9.7)	1 (3.2)	4 (12.9)
Bilateral breast cancer before 51	3	1 (33.3)	-	1 (33.3)
Breast and ovarian cancer	7	1 (14.3)	-	1 (14.3)
Medullary breast carcinoma	14	3 (21.4)	-	3 (21.4)
Breast cancer families (HBC)				
2 breast cancer cases	25	5 (20.0)	2 (8.0)	7 (28.0)
≥3 breast cancer cases	35	6 (17.1)	1 (2.9)	7 (20.0)
Total	60	11 (18.3)	3 (5.0)	14 (23.3)
Breast and ovarian cancer families (HBOC)				
1 breast cancer and 1 ovarian cancer	5	1 (20.0)	2 (40.0)	3 (60.0)
≥3 breast and ovarian cancer cases	27	14 (51.9)	2 (7.4)	16 (59.3)
Total	32	15 (46.9)	4 (12.5)	19 (59.4)
Ovarian cancer families (HOC)				
≥2 ovarian cancer cases	4	1 (25.0)	1 (25.0)	2 (50.0)

HBC, hereditary breast cancer; HBOC, hereditary breast and ovarian cancer; HOC, hereditary ovarian cancer.
